# Long-term mid-facial growth of patients with a unilateral complete cleft of lip, alveolus and palate treated by two-stage palatoplasty: cephalometric analysis

**DOI:** 10.1007/s00784-016-1949-2

**Published:** 2016-09-16

**Authors:** I. F. P. M. Kappen, G. K. P. Bittermann, R. M. Schouten, D. Bittermann, E. Etty, R. Koole, M. Kon, A. B. Mink van der Molen, C. C. Breugem

**Affiliations:** 1 Department of Plastic Surgery, Wilhelmina Children’s Hospital, Lundlaan 6, PO Box 85090, 3508 AB Utrecht, The Netherlands; 2 Department of Maxillofacial Surgery, University Medical Centre Utrecht, Heidelberglaan 100, PO box 85500, 3508 AB Utrecht, The Netherlands; 3 Department of Methodology and Statistics, University of Utrecht, Padualaan 14, 3584 CH Utrecht, The Netherlands

**Keywords:** UCLP, Two-stage palatoplasty, Cephalometry, Facial growth, Long-term results

## Abstract

**Objectives:**

The aim of this study is to evaluate long-term facial growth in adults previously treated for an isolated unilateral complete cleft lip, alveolus and palate by two-stage palatoplasty.

**Materials and methods:**

Unilateral cleft lip and palate (UCLP) patients of 17 years and older treated by two-stage palatoplasty were invited for long-term follow-up. During follow-up, lateral cephalograms were obtained (*n* = 52). Medical history was acquired from their medical files. Outcome was compared to previously published normal values and the Eurocleft study.

**Results:**

Soft and hard palate closure were performed at the age of 8 (SD 5.9) months and 3 (SD 2.2) years, respectively. The mean maxillary and mandibular angle (SNA, SNB) were 74.9° (SD 4.2) and 75.8° (SD 3.8). Maxillary and maxillomandibular relationships (SNA, ANB) were comparable to all Eurocleft Centres, except for Centre D. We observed a significantly steeper upper interincisor angle compared to the Eurocleft Centres.

**Conclusions:**

This study describes the long-term craniofacial morphology in adults treated for a UCLP with hard palate closure at a mean age of 3 years. The mean maxillary angle SNA and mandibular angle SNPg were comparable to previous studies both applying early and delayed hard palate closure. The observed upper incisor proclination is likely caused by orthodontic overcorrection in response to the unfavourable jaw relationships. No clear growth benefit of this protocol could be demonstrated.

**Clinical relevance:**

The present study shows the long-term craniofacial morphology of UCLP adults after the Utrecht treatment protocol which includes two-stage palate closure.

## Introduction

Mid-facial growth is an important outcome measure when evaluating cleft lip and palate treatment. In patients with a unilateral cleft lip and palate (UCLP), normal maxillary growth is often impeded, resulting in a relative retrusion of the mid-face [1–3]. As untreated cleft patients often show a normal mid-facial growth potency, iatrogenic changes induced by the surgical treatment are likely the greatest cause for maxillary hypoplasia [3–6]. Delaying hard palate closure is therefore believed to minimize mid-facial growth interference as a larger portion of maxillary growth is already established. Nevertheless, delayed hard palate closure may in turn lead to less favourable speech results. To circumvent this dilemma, a two-stage approach was firstly introduced in the 1950s [7]. It was initially believed that early soft palate repair allows for adequate speech development while secondary delayed palate repair reduces the degree of growth restriction. In addition, the soft palate closure is thought to approximate the palatal shelves, avoiding the need for extensive palatal dissection at palate closure [8]. However, later studies investigating the potential mid-facial growth benefit of delayed hard palate closure showed contradictory results [4, 9]. The correlation between timing of closure and subsequent craniofacial growth therefore seems to be less evident. Other factors than timing of surgery are of influence and should be taken into account [4, 9].

Because the effects of surgery become increasingly apparent as patients mature [3], long-term assessment is essential to make a comprehensive evaluation of a treatment protocol and to identify the different factors affecting facial growth. So far, few long-term studies are available.

The objective of the present study is to evaluate and compare long-term mid-facial growth after two-stage palatoplasty in an adult group of UCLP patients with hard palate closure at the age of 3 years. Evaluation will be carried out by cephalometric analysis and results will be compared to the previously described treatment protocols, including those of the Eurocleft study.

## Methods

### Patients

Patients were selected from our cleft database at the Wilhelmina Children’s Hospital. We analysed all medical files of cleft patients that had been invited for a long-term assessment of their treatment (*n* = 148). This long-term multidisciplinary assessment was implemented since 2008 for all cleft patients of 17 years and older, who had cleft treatment at our hospital. Each medical file was scanned for surgical data and medical history, including the type of cleft. Only patients with a complete cleft lip and palate, including a complete cleft of the alveolus were considered for analysis. Furthermore, cleft repair had to comprise two-stage palatoplasty performed by one of the two surgeons of the Wilhelmina Children’s Hospital at that time. Patients with additional anomalies (*n* = 4), Simonart’s bands (*n* = 1), non-Caucasian ethnicity (*n* = 6), partial treatment elsewhere (*n* = 34), incomplete information regarding the timing of surgery or treatment according to a different protocol (*n* = 25) were excluded from the present study.

Out of the 148 patients that were invited for a last follow-up, 78 met the inclusion criteria for this study. From this group, 52 patients eventually attended follow-up (67 %). Out of the 26 patients that did not attend, 9 indicated that they were not interested in follow-up or unable to attend. The remaining patients were lost to follow-up either due to non-response or incorrect contact details (*n* = 17, 22 %). Factors that may have contributed to this loss are the lack of electronic files and the lack of standard follow-up after completion of the orthodontic treatment (before 2000). Lastly, the hospital changed location in 1999, which also might have led to the loss of up-to-date contact details.

The follow-up consultations took place between 2008 and 2014. Patient characteristics are described in Table 1. In order to ensure that the attended group (*n* = 52) was representable for the whole UCLP group (*n* = 78), we compared the treatment variables of the attended with the non-attended group in Table 1. We did not observe any significant differences between the two groups in terms of surgical timing or incidence of secondary surgeries.

**Table 1:** Patients’ characteristics baseline table

Patients’ characteristics		Followed up *n* = 52	Not followed up *n* = 26	*p* value*
Gender	Male (%)	37 (71.2 %)	26 (69.2 %)	0.861
Cleft side	Left (%)	32 (61.5 %)	10 (38.5 %)	0.269
Lip closure	Median age in months (IQR)	6.0 (4–7)	7 (4–9)	0.155
	Mean age in months (SD)	5.7 (2)	7 (3)	0.115
Soft palate closure	Median age in months (IQR)	5.0 (3–11)	6 (4–10)	0.995
	Mean age in months (SD)	7.8 (6)	6.9 (4)	0.481
Hard palate closure	Median age in months (IQR)	33.5 (25–44)	38 (38–59)	0.405
	Mean age in months (SD)	40 (26.4)	47 (31)	0.384
Pharyngoplasty	Total performed (%)	22 (42 %)	6 (23 %)	0.095
Orthognathic surgery	Total performed (%)	11 (21 %)	4 (15 %)	0.542
Fistulas	Clinical significant (%)	14 (27 %)	5 (19 %)	0.569

Description of patient characteristics

*IQR* interquartile range

*p value <0.05 was regarded as significant; chi-square tests, independent sample *t* tests and Mann-Whitney *U* tests were applied where relevant

### Surgical and orthodontic protocol

Surgeries were performed by two plastic surgeons from the Wilhelmina Children’s Hospital specialized in cleft lip and palate surgery. The Utrecht treatment protocol used is summarized in Table 2. The mean age of patients at each surgical intervention are shown in Table 1.

**Table 2:** Overview of the treatment protocols

		Pre-surgical orthopaedics	Lip closure	One-stage palate closure	Soft palate closure	Hard palate closure	Bone grafting
Utrecht		No	5–6 months (Millard technique)		7–9 months (Perko technique)	3 years (Von Langenbeck)	11 years
Eurocleft study	A	Yes	3–4 months (Millard, Skoog)		9–15 months (Von Langenbeck, Perko, Wardill, Kriens)	9 years	9 years
	B	No	2 months (Tennison + vomer plasty)	22 months (Wardill Pushback)			9 years
	C	No	<6 months (variation of methods + timing)	12 months (various methods and timing)			9 years
	D	Yes	<6 months (variation of methods + timing)	Within 2 years (various methods and timing)			9 years
	E	No	3 months (Millard + vomer plasty)	18 to 20 months (mod. Von Langenbeck)			9 years
	F	Yes	4–6 months (mod. Skoog, Tennison-Randall)	12 months (Veau-Wardill-Kilner			4–6 months
Nijmegen		Yes	6–8 months (Millard)		12–14 months (mod. Von Langenbeck)	9–11 years (Boyne and Sands)	9–11 years
Gothenburg		–	3 months		7.5 months (subperiostal flaps)	8 years	8 years

Overview of the treatment protocols applied in previous long-term studies [2, 12], including the Eurocleft study [11]. The surgical technique is indicated between brackets. For a more detailed description of the Utrecht protocol see Table 1

*Mod* modified

Patients presenting with persisting velopharyngeal insufficiency despite additional speech therapy directed at improvement of velum mobility, were offered a subsequent pharyngoplasty. Speech enhancing surgery was performed according to the modified Honig technique [10].

Orthodontic treatment was carried out by our orthodontist (E.E.), also experienced in cleft care. Pre-surgical plates were only applied in case of significant feeding problems or tongue thrust during infancy and therefore not used as standard. Orthodontic treatment was started at least 6 months before alveolar bone grafting and resumed 6 months after the surgical intervention. Removable appliances were used to widen the maxilla establishing a normal transversal occlusion prior to alveolar bone grafting. A fixed orthodontic appliance was used to correct the vertical plane of the central incisors. The transverse expansion was maintained for at least 6 months after alveolar bone grafting. After these 6 months, the fixed appliances were reapplied to establish the best possible occlusion. Facial masks were not used in our protocol.

### Cephalometric analysis

**Fig. 1:**
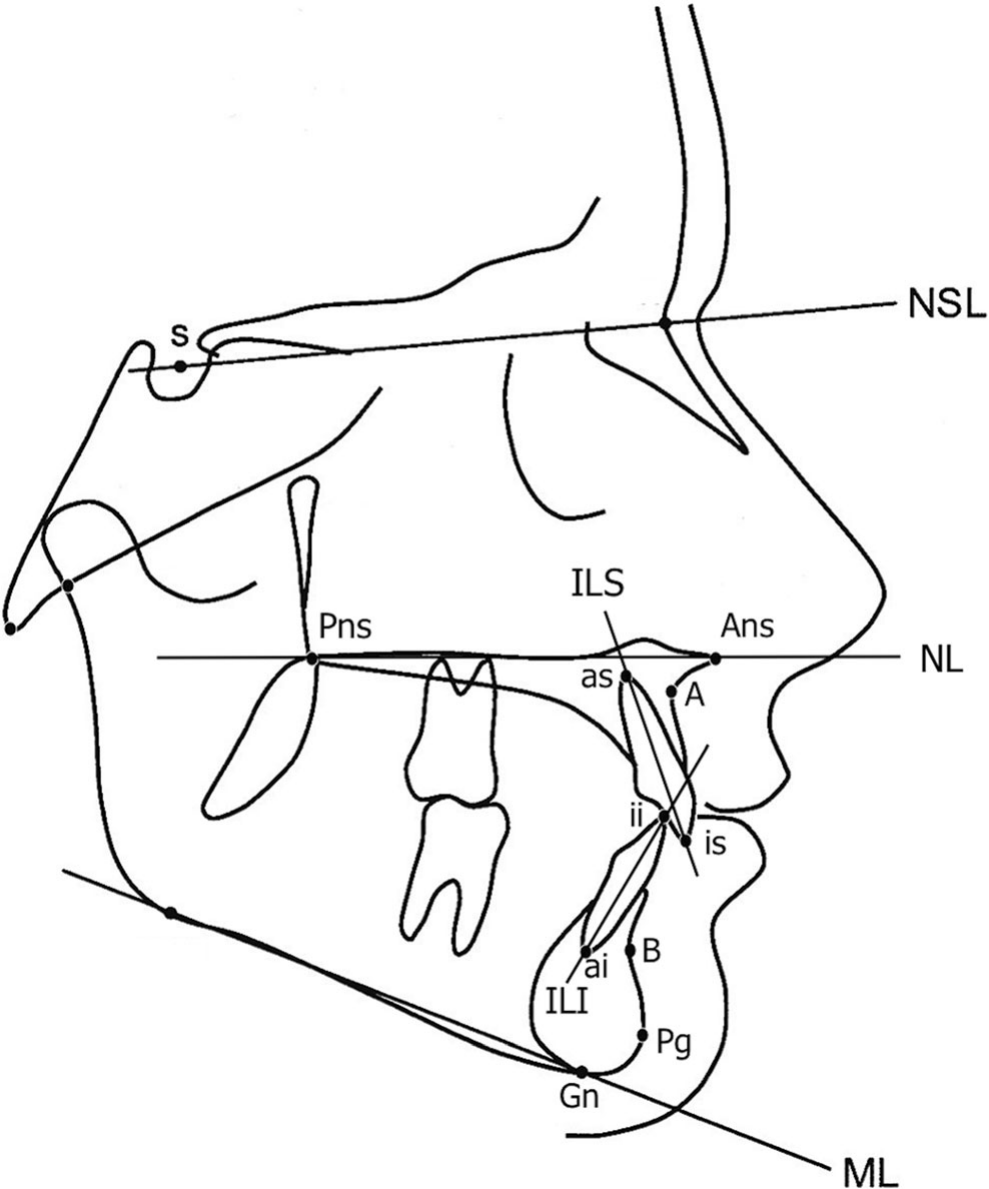
Skeletal reference points and reference lines. Reference points: *A* subspinal, deepest anterior point in the concavity of the anterior maxilla. *Ai* apex inferius, apex of the root of the most prominent lower central incisor. *Ans* anterior nasal spine, most anterior point of the anterioposterior profile of the upper jaw. *As* apex superius, apex of the root of the most prominent upper central incisor. *B* supramental point, deepest anterior point in the concavity of the anterior mandible. *Gn* gnathion, the most inferior point on the mandibular symphysis. *N* nasion, most anterior point of the frontonasal suture. *Pg* pogonion, most anterior point on the mandibular symphysis. *Pns* posterior nasal spine, most posterior point of the bony palate defined by the junction of the hard palate, the soft palate and the extension of the pterygomaxillary fissure. *S* sella, the centre of the sella turcica. Skeletal reference lines: *ILI* axis of lower incisors, line through *ai* and *as*. *ILS* axis of upper incisors, line through *as* and *is*; *ML* mandibular line, the tangent to the lower border of the mandible through the lowest point of the mandibular symphysis (*Gn*). *NL* nasal line or palatal plane, the line through *Ans* and *Pns*. *NSL* nasion-sella-line, line through the central sella (*point S*) and nasion (*point N*)

Standardized lateral cephalograms were obtained on the day of long-term follow-up. If the patient had a history of orthognathic surgery, the pre-operative cephalogram was analysed in this study. The Orthophos XG 3^®^ (Sirona group, Salzburg, Germany) was used for imaging. Each cephalogram was made in natural head position with teeth occluded. Images were stored as a DICOM file and subsequently exported to Viewbox 4.0, a software program for cephalometric analysis (dHAL Software^®^, Athens, Greece, 2014). Each image was rescaled before analysis. The first stage of analysis involved determining 12 landmark points on each cephalogram. These landmark points were used to determine 5 reference lines, from which 10 angles and 1 ratio variable (Fig. 1) could be calculated. The calculated values included the angles used by the Eurocleft study, Nollet et al. and Friede et al. [2, 11, 12]. In order to calculate the inter- and intra-observer agreement, all cephalograms were scored twice by a maxillofacial trainee (G.B.) and a medical student (I.K.) under the same conditions, with an interval of at least a week. The cephalometric variables were compared to normal values described by Thilander et al. [13].

### Statistical analysis

Statistical analysis was carried out using SPSS (IBM SPSS Statistics 20.0, IBM Inc., NY, USA) and RStudio (^©^2009–2015 RStudio, Inc., Boston, USA). A *p* value below 0.05 was considered as significant. The chi-square test or independent sample *t* test was used to compare patient groups where relevant. The Mann-Withney *U* test was used to compare median values.

Cephalometric analysis was repeated multiple times in order to calculate the inter- and intra-rater variability. For statistical analysis, we used the mean value of the angles obtained during the different scoring sessions. The pre-operative cephalogram was missing in 5 of the 11 patients that had orthognathic surgery (9.6 % of total). To prevent a possible bias, we predicted and imputed the values for these missing cephalograms using a multiple regression analysis. Known values of baseline characteristics, surgical history, and the timing of each surgical procedure were used as predictors in the model. The procedure was repeated five times, resulting in 5 separate datasets. From these datasets, a pooled mean estimate was derived for each of the missing cephalometric values.

The observed mean cephalometric values were subsequently compared to normal values [13] and the mean values of previous studies including those of the Eurocleft study [2, 11, 12]. The one-way ANOVA test was used to compare all means [2, 11, 12]; contrast tests were used to calculate the mean differences between each study and to determine the statistical significance of these differences. Given the multiple comparisons, a modified Bonferroni correction was applied to calculate an adjusted *p* value after each one-way ANOVA test in order to maintain higher statistical power [14].

In the study of Thilander et al., normal values were originally described for men and women separately [13]. In order to maintain statistical power, a pooled mean value for both sexes was calculated and used for comparison according to the method described above. The pooled standard deviations were calculated using the following formula: pooled sd = √ ((S1 × (n1 − 1)) + (S2 × (n2 − 1))/((n1 − 1) + (n2 − 1)) (S1 = variance for women (variance = sd^2), n1 = total number of women, S2 = variance for men (variance = sd^2), n2 = total number of men ).

The interclass correlation coefficient (ICC) was used to calculate the intra-observer and inter-observer reliability of the cephalometric analysis. ICC values above 0.75 are considered as excellent, values between 0.40–0.74 are fair to good and values below 0.40 are considered as poor [15]. In addition, reliability was measured by calculating the difference, 95 % limits of agreement, and error of the method as described by Bland and Altman [16]. Error of the method is therefore calculated using the following equation: √(sd^2/n).

### Ethical approval

The Medical Ethics Committee of the University of Utrecht approved the protocol (14/416) and methods used for this study which was performed in accordance with the 1964 Declaration of Helsinki and its later amendments.

## Results

### Measurement reliability

The intra- and inter-observer analyses demonstrated good intra-class correlation coefficients for all obtained cephalometric values, varying from 0.795 to 0.977 and from 0.725 to 0.983, respectively (Table 3). The inter-observer differences and limits of agreement were greatest for the dentoalveolar values and small for the SNA, SNB and ANB values (Table 3).

**Table 3:** Agreement of the cephalometric values

	Paired differences		95 % CI of the difference	95 % limits of agreement	ICC	Error of the method
	Mean	SD				
Intra-observer agreement scoring sessions 1 and 2 of rater 1						
SNA (°)	0.46	1.32	0.03–0.88	−2.13–3.06	.977	0.21
SNB (°)	0.13	1.19	−0.26–0.51	−2.21–2.46	.977	0.19
ANB (°)	0.46	1.33	0.04–0.89	−2.14–3.06	.963	0.21
NSL-NL (°)	−1.3	2.45	−2.11–-0.53	−6.13–3.48	.851	0.39
SNPg (°)	0.24	1.11	−0.12–0.59	−1.9–2.40	.979	0.18
NSL-ML (°)	−0.72	1.40	−1.34–0.10	−4.52–3.08	.964	0.31
NL-ML (°)	−0.19	1.45	−0.66–0.27	−3.04–2.64	.913	0.23
ILS-ILI (°)	−1.39	5.48	−3.15–0.37	−12.14–9.35	.899	0.87
ILS-NL (°)	2.25	4.07	0.92–3.60	−5.72–10.23	.864	0.66
ILS-NA (°)	0.12	1.19	−0.26–0.51	−2.21–2.46	.923	0.19
Intra-observer agreement scoring sessions 1 and 2 of rater 2						
SNA (°)	−0.13	1.05	−0.47–0.21	−2.19–1.93	.977	0.17
SNB (°)	−0.16	0.87	−0.43–0.12	−1.85–1.54	.988	0.14
ANB (°)	−0.03	0.58	−0.21–0.16	−1.17–1.12	.986	0.09
NSL-NL (°)	−0.10	3.15	−1.1–0.90	−6.28–6.07	.795	0.50
SNPg (°)	−0.09	0.82	−0.36–0.17	−1.71–1.51	.989	0.13
NSL-ML (°)	0.46	1.57	−0.04–0.96	−2.61–3.53	.980	0.25
NL-ML (°)	0.04	3.21	−0.99–1.06	−6.25–6.32	.916	0.51
ILS-ILI (°)	−0.15	5.41	−1.88–1.58	−10.75–10.45	.910	0.86
ILS-NL (°)	0.38	4.20	−0.96–1.72	−7.85–8.6	.950	0.66
ILS-NA (°)	0.19	2.95	−0.75–1.13	−5.59–5.97	.962	0.47
Inter-observer agreement scoring session 1 between rater 1 and rater 2						
SNA (°)	−0.46	1.96	−1.11–0.18	−4.30–3.37	.920	0.32
SNB (°)	0.21	1.76	−0.37–0.79	−3.23–3.65	.946	0.28
ANB (°)	−0.70	0.99	−1.03–−0.375	−2.65–1.25	.940	0.16
NSL-NL (°)	1.55	3.57	0.38–2.73	−5.44–8.54	.747	0.58
SNPg (°)	0.84	1.69	−0.47–0.64	−3.23–3.4	.900	0.27
NSL-ML (°)	−1.05	0.43	−1.92–−0.18	−6.23–4.14	.895	0.43
NL-ML (°)	−3.35	2.92	−4.24–−2.47	−9.09–2.37	.874	0.44
ILS-ILI (°)	−2.54	4.65	−4.53–−0.54	−11.7–6.64	.848	0.76
ILS-NL (°)	5.4	4.19	4.06–6.92	−2.77–13.65	.908	0.67
ILS-NA (°)	1.62	3.48	0.47–2.76	−5.2–8.44	.930	0.56

Bland Altman, ICC agreement and Dahlberg formula for calculating the inter- and intra-observer agreement of the cephalometric analysis. All ICC values were statistically significant (*p* value <0.05).﻿ *ICC -﻿* interclass co﻿rrelation coefficient. *95% CI - *95% confidence interval

### Comparison to normal values

**Fig. 2:**
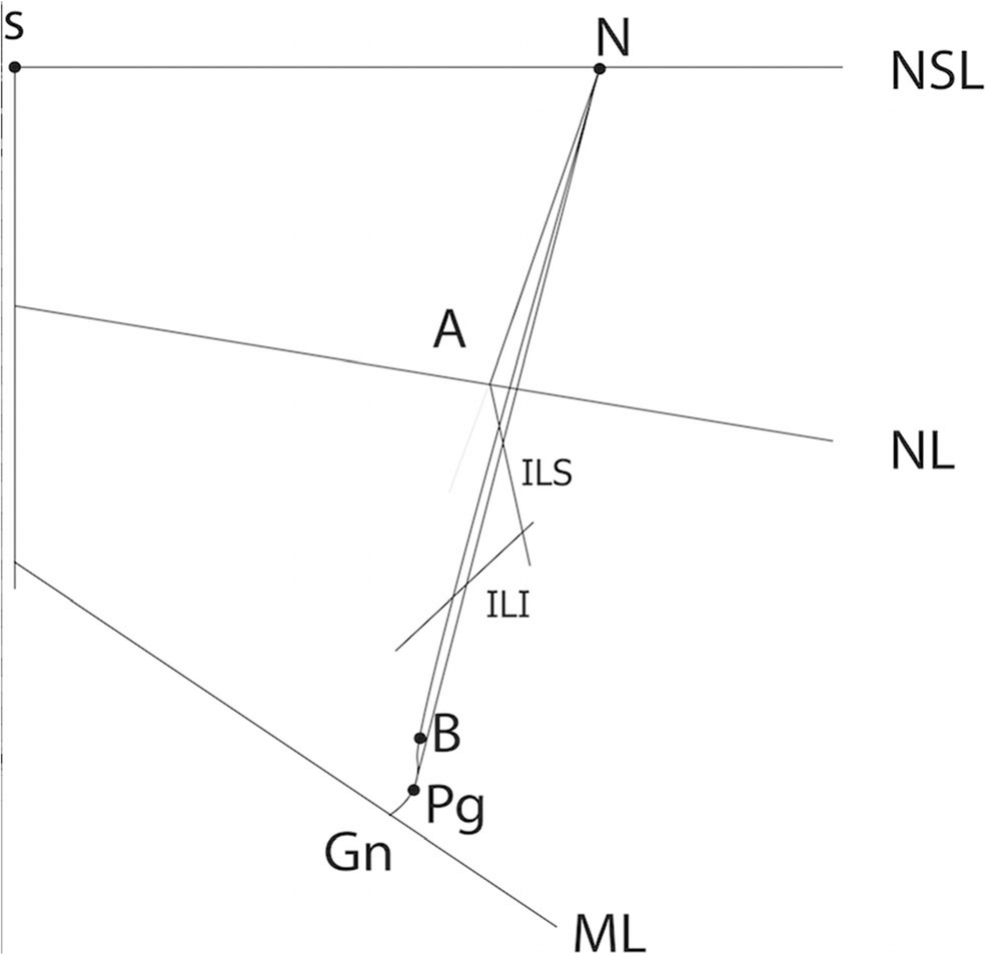
Composite tracing based on the mean cephalometric values of the studied cohort. Reference points are explained in the legend of Fig. 1

The mean cephalometric values were compared to the pooled normal values in Table 4 and shown in Fig. 2. The maxillary angle (SNA) and the maxillomandibular angle (ANB) were significantly smaller and negative compared to the normal population, indicating maxillary hypoplasia. Both the vertical maxillary inclination and mandibular inclination were significantly increased (NSL-NL, NSL-ML). We also observed an increase of the ILS-NA angle and more obtuse inter-incisor angle (ILS-ILI).

**Table 4:** Comparison of the presently obtained values to normal values

	Male UCLP patients	Female UCLP patients	UCLP patients	Pooled normal values	Mean difference total group*	95 % CI^a^
	Mean (SD)	Mean (SD)	Mean (SD)	Pooled mean (pooled SD)		
Mean age	*20.4* (*3.5*)	*21.6* (*3*)	*21* (*3.4*)			
Median age	*20*	*21*	*20*			
Maxillary values						
SNA (°)	74.3 (3.3)	75.8 (5.1)	74.9 (4.19)	83.0 (3.38)	−8.1*	−9.89–−6.31
NSL-NL (°)	8.7 (4.2)	8.7 (4.1)	8.5 (3.90)	6.3 (2.6)	0.56*	3.81–0.84
Mandibular values						
SNB (°)	75.6 (3.4)	75.8 (3.8)	75.7 (3.73)	81.1 (3.31)	−5.4*	−6.99–−3.81
SNPg (°)	76.5 (3.5)	76.1 (3.6)	76.5 (3.62)	82.5 (2.5)	−6.0*	−7.70–−4.30
NSL-ML (°)	34.8 (5.9)	37.0 (6.8)	35.4 (6.35)	28.5 (4.44)	4.33*	9.48–1.31
Maxillomandibular relations						
ANB (°)	−1.4 (2.6)	0.1 (2.7)	−0.9 (2.71)	1.8 (2.06)	−2.8*	−4.14–−1.45
NL-ML (°)	25.8 (5.6)	27.6 (5.7)	26.5 (5.84)	21.7 (4.98)	6.86*	4.30–9.42
Dentoalveolair values						
ILI-ILS (°)	127.9 (7.6)	128.9 (9.1)	128.2 (8.50)	133.7 (8.2)	−0.59*	−1.00–−0.12
ILS-NL (°)	111.0 (7.11)	109.2 (6.8)	110.2 (6.98)	108.6 (6.45)	1.56	−1.40–4.52
ILS-NA (°)	29.7 (6.3)	25.0 (7.7)	28.4 (7.13)	19.8 (6.40)	7.6*	4.53–10.67

Comparison of the presently observed cephalometric values to normal values described by Thilander et al. [13]. The location of each cephalometric point and angle is indicated in Fig. 1. A pooled mean and pooled standard deviation (methods) was calculated for both sexes calculated from the mean values for man and women at 19 years of age

*Mean difference is statistically significant, the 95% confidence interval for the mean difference ﻿does not contain the number 0.

^a^95 % CI—95 % confidence interval of total group

### Comparison to previously reported long-term values after cleft treatment

**Fig. 3:**
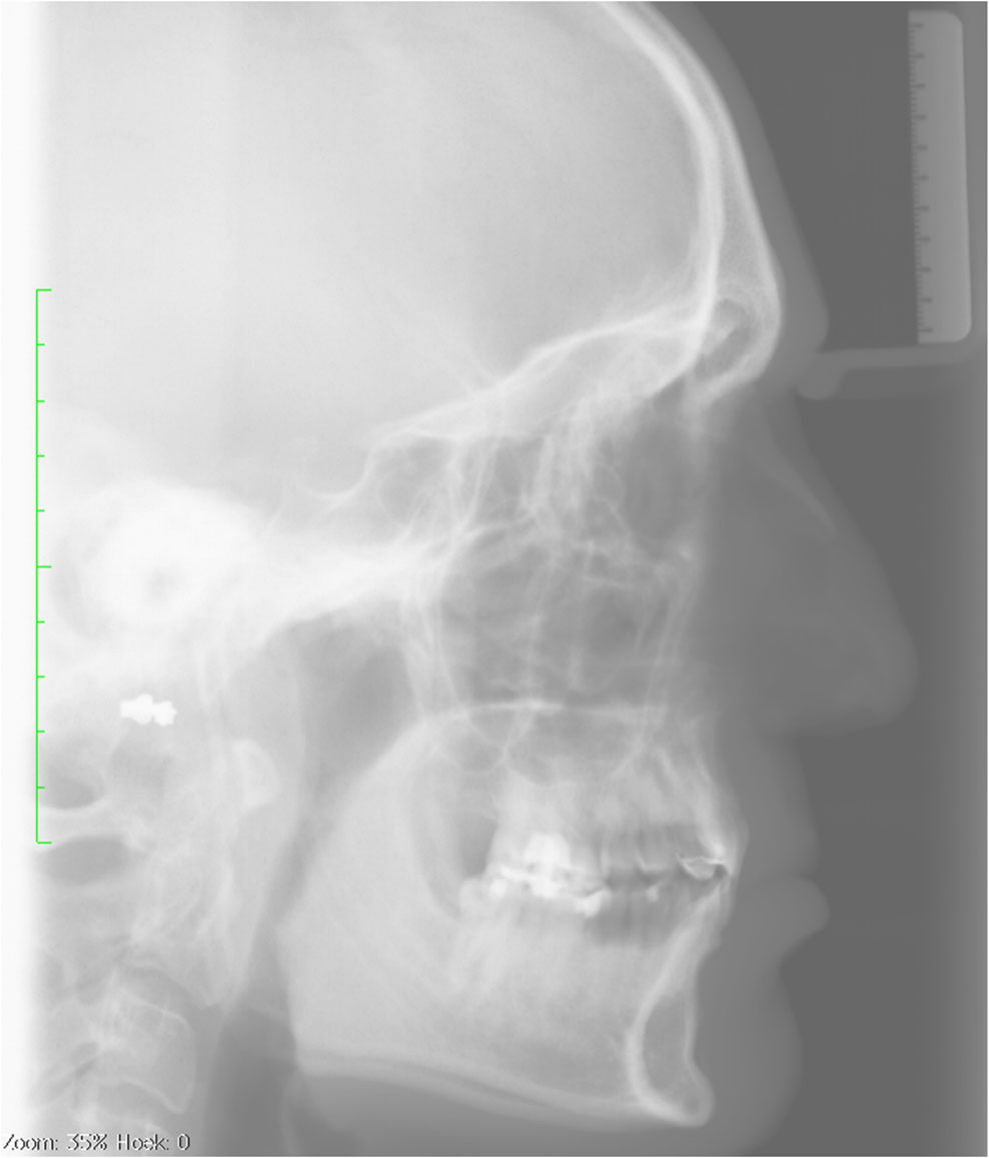
A lateral cephalogram made in natural head position with teeth occluded, scaled and exported to Viewbox

Our mean cephalometric values are compared to previous studies in Table 5 and presented in Fig. 2. An example of a cephalogram can be seen in Fig. 3.

**Table 5:** Comparison of cephalometric values with previous studies

	Utrecht mean (SD)	Eurocleft					Gothenburg	Nijmegen
		A	B	D	E	F		
		Mean (SD)					Mean (SD)	Mean (SD)
Mean age	21 (3.4)	17	17	17	17	17	18.9 (0.4)	18 (1.2)
Number of patients	52	24	26	24	30	20	50	37
Maxillary values								
SNA (°)	74.9 (4.19)	74.5 (4.4)	75.7 (5.1)	72.9* (*4.5*)	74.9 (3.7)	74.1 (4.1)	76.8* (*3.2*)	74.3 (4.5)
NSL-NL (°)	8.5 (3.90)	8.9 (4.1)	8.9 (4.0)	11* (*4.5*)	8 (3.6)	6.0* (*4.9*)	8.8 (3.2)	9.5 (3.6)
Mandibular values								
SNPg (°)	76.5 (3.62)	76.4 (4.9)	78.1 (4.1)	76.8 (4.4)	78.0 (3.6)	78.0 (4.4)	78.1* (*3.1*)	75.7 (4.7)
NSL-ML (°)	35.4 (6.35)	37.2 (5.9)	33.5 (6.0)	37.5 (4.9)	35.1 (5.5)	37.2 (5.8)	32.8* (*5.8*)	35.7 (6.9)
Maxillomandibular relations								
ANB (°)	−0.9 (2.71)	−0.1 (2.5)	−0.7 (2.4)	−2.2 (3.6)	−0.9 (2.2)	−2.4 (4.7)	−0.1 (2.5)	−0.4 (3.8)
Vertical dimensions								
N-Ans/N-Gn × 100	41.0 (2.5)	42.1* (*2.2*)	42.0 (2.2)	43.2* (*2.6*)	41.5 (2.4)	40.6 (1.9)	42.7* (*1.7*)	44.1* (*2.0*)
Dentoalveolair values								
ILS-NL (°)	110.2 (6.98)	95.8* (*5.8*)	93.4* (*5.4*)	90.0* (*7.1*)	94.2* (*6.2*)	94.1* (*10.4*)	103.6* (*5.6*)	111.0 (6.3)
ILI-ILS (°)	128.2 (8.50)	127.8 (13.4)	138.7* (*9.8*)	142.5* (*8.8*)	136.6* (*7.2*)	137.5* (*10.6*)	–	131.7 (12)

Comparison of mean cephalometric values to previous long-term studies [2, 11, 12]. The locations of the specific points and angles are indicated in Fig. 1

*The 95 % confidence interval obtained from the contrast tests did not contain the number 0, and mean difference was statistically significant

Eurocleft Centre D showed a significantly less favourable mean SNA angle. [11] The NSL-NL angle was also increased. Mandibular angles were comparable to the present study (NSL-ML, SNPg). Vertical facial proportions were more harmonious in Centre D, Centre A and the Centre of Nijmegen. (N-Ans/N-Gns ×100 %).

The maxillary (SNA) and maxillomandibular (ANB) relationships of Centre A and of Nijmegen, both applying two-stage closure, were comparable to the current study. Further, the group treated according to the Gothenburg protocol showed better maxillary (SNA) and mandibular relationships (SNPg, NSL-ML) compared to this study.

All Eurocleft Centres showed significantly reduced angles between the upper incisor and palatal plane (ILS-NL) resulting in a more obtuse interincisor angle (ILI-ILS).

## Discussion

This study reports the long-term craniofacial morphology in UCLP adults after delayed hard palate closure performed at the age of 3 years. Compared to the general population, the present cohort observed mid-facial growth differences recognized as typical characteristics of individuals with a repaired unilateral complete cleft lip and palate (UCLP). [11, 12]. The maxillary and mandibular angles SNA and SNB were reduced and we observed a more retruded position of the maxilla in relation to the mandibula (ANB) (Table 4). Further, a more obtuse gonial angle and steeper mandibular plane (NSL-ML, NL-ML) was observed in the current cohort. General consensus is that these growth differences are both a direct and an indirect result of cleft palate surgery [3–5]. Scar tissue along the dental arch may also cause a significant deviation of the dentoalveolar process resulting in dental malocclusions and often retroclined incisors [17]. The observed mean ILS-NA angle and inter-incisor angle (ILI-ILS) deviated from the normal values, likely due to an increased upper incisor inclination as a result of orthodontic treatment, and a more retroclined position of point A, which is often seen in cleft individuals. We therefore found a significant growth impairment despite delaying the hard palate closure until the age of 3 and performing the osteoplasty relatively late.

When comparing our cephalometric values to previous long-term results, the observed maxillary (SNA) and maxillomandibular (ANB) relationships were similar to studies applying both one- (Eurocleft Centres B, E, F) and two-stage closure (Eurocleft Centre A, Nijmegen) [2 11, 12] (Table 5). Although there was a trend of increased maxillary retrusion in relation to the mandibula (ANB) in centres applying early palate closure (Centres D and F), this difference did not reach statistical significance. This lack of difference might however be related to the small patient groups, resulting in low statistical power [11]. It is questionable whether the timing of surgery contributed to this trend, as other one-stage centres of the Eurocleft study show relatively better outcomes (Centres B, E). The outcome of Center D may have been affected by the inconsistency of their protocol and participation of low volume surgeons. Similarly, the moderate results of Centre F likely resulted from the implementation of primary bone grafting during lip closure (Table 2) [18–20]. A review of long-term studies after two-stage closure found that disappointing growth outcomes after two-stage closure can often be explained by surgical variations or other factors within the treatment protocol [21]. Although some studies found better growth results after delayed closure [22], a direct correlation between timing of closure and growth outcome seems to be lacking and factors other than timing should be taken into account [9, 23].

The amount and location of scar tissue may have a greater influence on mid-facial growth than the specific age of hard palate closure during the first decade of life. Several studies have attributed the decreased maxillary growth after a two-stage protocol to increased scar tissue formation [24, 25]. Scar tissue around the sutures, such as the vomero-premaxillary suture, can restrict the forward and downward expansion of the maxilla [17, 26, 27]. A significant proportion of the final length of the maxilla is gained during the maxillary growth spurt. According to cephalometric analysis of the general population, this increase in growth velocity takes place at the age of 6–10 years in girls and 8–14 years in boys [13]. Delayed hard palate closure at the age of 3 years might therefore be too early as post-operative scar tissue can still interfere significantly with growth. This may also explain why Schweckendieck obtained such good results after hard palate closure at the mean age of 13 (range 8–22 years), after the previously described growth spurt [28]. The growth benefit of delayed hard palate closure may therefore only be achieved when closing at a significantly later age, when the greatest proportion of the final maxillary length is already achieved.

The extent of scar tissue formation is influenced by multiple factors such as surgical skill and experience, a patient’s inherent propensity for scar formation [3] and the amount of secondary surgeries. This study found a high incidence of revision cheiloplasty (46 %), pharyngoplasties (42 %) and fistulas (27 %) needing surgical closure. Previous studies identified a significantly higher rate of secondary procedures (including pharyngoplasties) in patients with poor growth outcomes or patients needing orthognathic surgery [24, 29, 30]. Similarly, the worst scoring Eurocleft Centre D had the highest number of surgeries per patient (6.0 surgeries compared to 4.4 and 4.8 in Centres E and A, respectively) [31]. Extensive fibrosis following primary surgery might contribute to the need for secondary surgeries (a pharyngoplasty due to a rigid velum) as well as to the development of maxillary hypoplasia. The observed correlation between the number of surgeries and maxillary growth outcome may therefore not be causal. Attempt to minimize scar formation during primary surgery should be considered an important goal. For this reason, expertise, skill and caseload of the surgeon might also have a great influence on long-term results after cleft treatment. Unfortunately, the actual contribution of each surgical and non-surgical treatment factor to impaired mid-facial growth is difficult to quantify because of their interplay and collective action.

Orthodontic treatment to optimize dental occlusion is an important non-surgical treatment factor. Of significance in the present results, the mean inter-incisor angle differed substantially from all Eurocleft Centres except for Centre A [11]. (Table 5) The inter-incisor angle in our group was however still significantly smaller compared to normal values. (Table 4) This smaller inter-incisor angle is likely caused by a more pronounced upper incisor proclination. Liao et al., reported previously that early closure of the palate resulted in more retroclined incisors [32]. However, these incisors tend to procline over time due to a dento-alveolar compensation mechanism in response to the unfavourable arch relationships [32]. Further, as the von Langenbeck procedure limits the amount of scar tissue adjacent to the anterior alveolus, this technique is believed to facilitate the compensatory anterior incisor’s adjustment [19, 32]. Orthodontic treatment may in turn enhance this inclination, resulting in overcompensation and steep incisor angles. The underlying cause for this compensation, maxillary hypoplasia, however remains present. Our findings may therefore be explained by the rigid orthodontic treatment that was followed in Utrecht, while maintaining a conservative approach towards orthognathic surgery. According to Good et al., the decision for performing a maxillary advancement is based on subjective criteria and possibly influenced by cultural differences in patient expectations and surgical preferences [29]. The decision to perform orthognathic surgery will therefore vary amongst centres. The studied patients were possibly reluctant to undergo orthognathic surgery, which may have been influenced by the opinion of our orthodontists at that time. Of note, the 95 % limits of agreement were more dispersed for the dentoalveolar values making the error of the method larger for these measurements.

In summary, heterogeneity of treatment protocols and multifactorial influences on treatment outcomes pose a challenge for clinical cleft lip and palate research. Focus has previously been on the timing of cleft closure, however as these results suggest surgical, non-surgical and patient factors all play a role.

### Limitations

In this retrospective study, quality of data partly depends on the accuracy of medical records. Looking back as far as 30 years, some data could not be retrieved and the cases within this study were not consecutive. This increases the risk of selection bias. Further, as cephalometric normal values for the Dutch population are lacking, Swedish normal values were used as they are geographically the most closely related. However, mid-facial growth patterns may vary amongst Caucasian populations [33] and we cannot ensure that mid-facial growth in the Swedish population exactly relates to the growth patterns of the Dutch.

An uncertainty was implemented in the data by using the technique of multiple imputation to insert missing values (*n* = 5). The currently described mean values were therefore based on pooled estimates. However, as many predictors were used in the imputation model and the procedure was repeated five times before calculating the pooled estimates, this uncertainty could be considered negligible. Removing five cases that contained missing values would have further reduced the statistical power and might also have introduced a bias. In addition, the patient cohorts described in the previous studies and by Thilander et al., were sometimes small (varying from 20 to 50) [2, 11–13]. and therefore already had a decreased statistical power.

Despite a possible difference in cephalometric values between male and female groups, we did not analyse these groups separately as it would again affect the power of this study. The mean values for males and females are however separately reported in Table 4.

Although the patient characteristics of those that did or did not attend follow-up was not significantly different, (Table 1), patients with a more intensive treatment or less favourable outcome were possibly more inclined to attend follow-up. This might have led to an underestimation of results.

## Conclusion

This study describes the long-term craniofacial morphology in adults treated for an UCLP with hard palate closure at a mean age of 3 years. The mean maxillary angle SNA and mandibular angle SNPg were comparable to previous studies both applying early and delayed hard palate closure. The observed upper incisor proclination is likely caused by orthodontic overcorrection in response to the unfavourable jaw relationships. No clear growth benefit of this protocol could be demonstrated.

The cause for impeded mid-facial growth after cleft surgery is multifactorial. In future treatment protocols, emphasis should not solely be on the timing of surgery but also on the minimization of palatal scar tissue in order to reduce growth disturbances later in life. The high rate of secondary surgeries and resulting scar tissue formation is possibly one of the contributing factors to the moderate growth results in our group.
